# The Difficulties in Demonstrating That Aflatoxin Reduction Improves Stunting in Developing World Regions

**DOI:** 10.3390/toxins18010032

**Published:** 2026-01-09

**Authors:** Paul C. Turner, Erica Phillips

**Affiliations:** 1 School of Public Health, University of Maryland, College Park, MD 20742, USA; 2 Ross and Carol Nese College of Nursing, The Pennsylvania State University, State College, PA 16802, USA; elp28@cornell.edu

**Keywords:** aflatoxin, infant, stunting, growth, exposure, aflatoxin-lysine, aflatoxin-albumin

## Abstract

Aflatoxins are highly toxic secondary metabolites that contaminate dietary staples in many developing world regions, with hundreds of millions of people estimated to be chronically exposed. In this review, we summarize the evidence about AF exposure assessment and its relationship to stunting. Despite multiple attempts, this question has eluded a strong scientific conclusion due to the nature of the toxin and exposure, the disparate methods used for assessment, and the ethical difficulties of studying a toxin in low-resource settings. We highlight current challenges in defining these relationships, how this has reduced the ability to draw conclusions in this area, and approaches to overcome these to advance the field. Current reviews tend to report mixed associations, but typically lack critique of the study designs, and a limited understanding of patterns of aflatoxin exposure coupled with a probable variable threshold for effect. We highlight the potential diverse patterns of AF exposure over time and how that may influence study design to address this critical public health issue.

## 1. Introduction

Malnutrition refers to both the excesses and the deficiencies in nutrient intake, leading to an imbalance in growth and/or function. Undernutrition in children is characterized by anthropometry, such as stunting, wasting, underweight, and micronutrient deficiencies [1]. Stunted infants and children have a low height (or length) for age (HAZ or LAZ). Here, the term HAZ will be used throughout. Being stunted is defined as a HAZ of more than two standard deviations below the World Health Organization (WHO) median, typically expressed as a HAZ of <−2; while extreme stunting has a HAZ of <−3. The WHO describes stunting as the consequence of chronic or recurrent undernutrition, usually associated with poverty, poor maternal health and nutrition, frequent illness, and/or inappropriate feeding and care in early life. Stunting is more than just being shorter than average, and a low HAZ is associated with children and then adults failing to reach their physical and cognitive potential; specifically, the condition is associated with a higher risk of morbidity and mortality [2,3,4,5,6]. Globally, 14–17% of child deaths under 5 years are associated with stunting, and child undernutrition remains a significant public health concern [1,4,5,6,7,8,9,10,11] affecting about 148 million children under 5 years of age [1].

For several decades, the focus on mitigation of early life stunting has been on improved infant and young child feeding interventions (with or without micronutrient supplementation), and/or water, sanitation, and hygiene interventions, and while of value, especially to mitigate acute malnutrition, they appear to only provide modest improvements in stunting [12,13,14,15,16,17]. This lack of impact between well-established risk factors and efficacy of intervention to reduce stunting with strong and meaningful mitigation approaches suggests that other factors contribute to poor linear growth. One contribution that is frequently overlooked is the putative role of mycotoxins and, in particular, aflatoxins [18,19,20]. The aim of this review is to explore the potential role of aflatoxin in early life growth by; (a) introducing key concepts from several epidemiological studies, (b) illustrating the complexity of exposure assessment for aflatoxin, (c) highlighting a possible threshold of exposure for stunting, and (d) indicating complex patterns of exposure to aflatoxins in the first few years of life. Combined, these observations suggest a difficulty in demonstrating that aflatoxin interventions will lead to improved infant growth.

## 2. Mycotoxins

Under the right conditions of temperature and humidity, fungal contamination of foods by some species of *Aspergillus*, *Fusarium*, and *Penicillium* can lead to the production of a wide range of poisons known as mycotoxins [21,22,23]. Among thousands of these secondary metabolites, a handful contaminate key dietary staples at levels of contamination, frequency of contamination, and regularity of consumption to be of a public health concern [19,22,24,25,26]. Mycotoxins of major concern include aflatoxins produced from *Aspergillus flavus* and *A. parasiticus*; fumonisins, deoxynivalenol, and zearalenone produced from various *Fusarium* species, and ochratoxin A produced by both *Aspergillus* and *Penicillium* species [21,22,23]. Aflatoxins and fumonisins are typically more frequent contaminants of corn (maize) in hot and humid climates such as sub-Saharan Africa, Central America, and tropical Asia. Aflatoxins can additionally occur at high concentrations in groundnuts (peanuts) in tropical regions [19,21,22,23]. For aflatoxins, both field growth and long-term storage contribute to the burden of contamination, while fumonisins are predominantly a field-produced toxin of maize [19,21]. The other mycotoxins mentioned are more prevalent in temperate climates, though mixtures of all these toxins are observed in biofluids in both temperate and tropical countries [27,28]. Mycotoxins tend to be resistant to processing, and their stability during cooking also contributes to dietary exposure [21,22]. More developed regions of the world tend to have both the regulation and the infrastructure to enforce relevant laws, while the required infrastructure in developing-world regions is lacking, especially in subsistence or small-hold farm settings. Thus, individuals who are particularly vulnerable face a combination of limited dietary variation, heavy reliance on one or two high-risk dietary staples, and no useful regulation that influences or protects against exposure.

## 3. Aflatoxins

**Figure 1:**
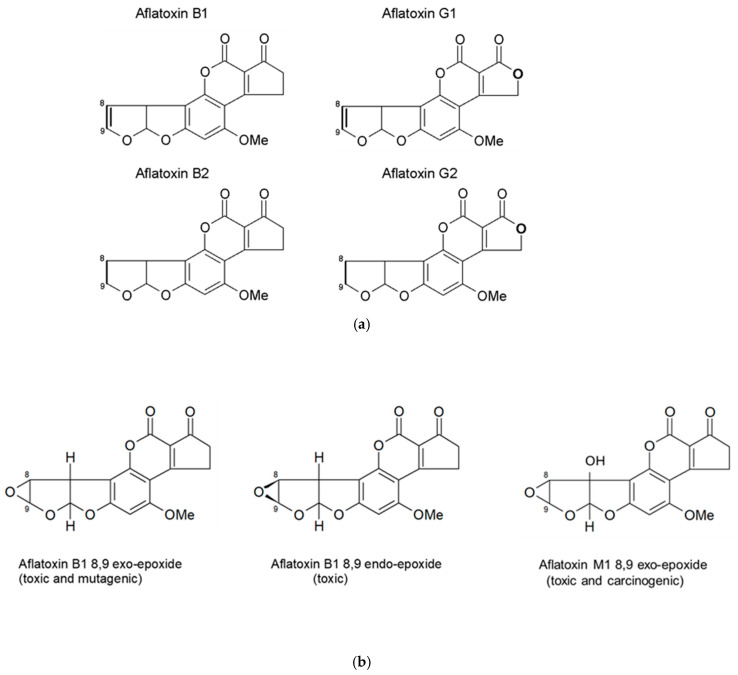
(**a**) Structures of the four naturally occurring aflatoxins. The -8,9- position is where the reactive epoxide can be readily formed across the double bond. (**b**) Structures of the aflatoxin B1 exo- and endo-epoxides, and aflatoxin M1 exo-epoxide.

Aflatoxins are a family of closely related poisons that occur naturally in certain grains and nuts, and include aflatoxin B1 (AFB1), AFB2, AFG1, and AFG2 (Figure 1a). AFB1 occurs most frequently and is the most toxic [19,21,22,29,30], and as such, AFB1 will be the focus of this review. Aflatoxin contamination of foods and subsequent human exposure vary by climate, season, geography, agricultural practice, and dietary behavior [18,19,31,32,33,34,35]. In many settings where crops are at risk of Aspergillus contamination and aflatoxin production, extended storage times in high humidity with insufficiently dried crops further increase contamination and risk of exposure [19,35].

Once ingested, AFB1 is metabolized by cytochrome P450 enzymes (CYPs) in many organs, as reviewed by [24,30,36,37], leading to an array of metabolites, including the toxic AFM1, and less toxic AFQ1 and AFP1; and two highly reactive epoxides, AFB1 exo-8,9-epoxide and AFB1 endo-8,9-epoxide (Figure 1b) [38,39]. The reactive epoxides cause toxicity via the formation of covalent adducts with multiple macromolecules, including nucleic acids and proteins [40,41]. The metabolite AFM1 can also undergo further transformation to form a reactive AFM1-epoxide, which is also toxic and carcinogenic [42]. Non-specific oxidative stress caused by aflatoxin biotransformation also contributes to toxicity [24].

Mechanism(s) of aflatoxin-induced stunting have been hypothesized and likely involve, in part, aflatoxin activation by CYPs to these damaging epoxides [18,19]. The main target organ for aflatoxin toxicity is the liver, and modulation of liver function may disrupt general nutritional homeostasis [18,43]. In addition, a reduction in insulin-like growth factor (particularly IGF-1) production and release by the liver, and/or other sites, may contribute to poor linear growth. Intestinal enterocytes contain CYPs that can also produce aflatoxin epoxides, and damage here leads to a reduction in both the functional surface of the intestine for nutrient absorption and the integrity of the intestine [44]. Given that early-life intestinal enteropathy, typically associated with gastrointestinal infections, is a well-established driver of infant stunting [45,46,47], it is reasonable to consider interactions of aflatoxin and intestinal infections that perhaps prolong the intestinal enteropathy, leading to a worsening of malnutrition. Aflatoxin also suppresses the immune system [48,49], including a demonstrated reduction in the protective secretory IgA in the saliva of Gambian children [50]; thus, aflatoxin may further contribute to this suggested interaction via a suppression of immune defenses. AFB1 also has epigenetic potential [51], with suggested modulation of genes such as IGF-1 that are associated with infant growth [52,53].

AFB1 is a proven human carcinogen [19,29], yet global efforts to reduce exposure remain limited. Exposures that critically impact infants often carry more power to drive action. However, despite multiple attempts, demonstrating strong scientific evidence for the suggested role of aflatoxin on poor linear growth has not been achieved. This is in part due to the variable nature of the toxin contamination and exposure profile, the disparate methods used for assessment, the limited sizes of many studies, and the ethical difficulties of studying this toxin in low-resource settings. Here, we highlight current challenges in defining these relationships, how this has reduced the ability to draw conclusions in this area, and approaches to overcome these to advance the field.

## 4. Aflatoxin Exposure Assessment

While several foods are potentially contaminated, the major foods causing aflatoxin exposure in high-risk settings typically involve one or two limited dietary sources: maize (corn) and/or groundnuts (peanuts); foods that are typically dietary staples and used as complementary feeding in many tropical world regions [19]. Robust and accurate exposure tools are essential to conduct epidemiological research and to assess the efficacy of mitigation efforts to reduce aflatoxin exposure [27,28,54,55]. Aflatoxin can be readily detected in food samples; however, food is a precious commodity, and thus, food sampling for toxins becomes problematic in many low-resource settings. In addition, aflatoxins are extremely heterogeneously distributed within foods, typically making exposure assessment imprecise unless sampling regimes are extensive. By contrast, the measurement of body fluids can capture the concentration of either the parent toxin (or a metabolite) more accurately, and thus, provide an improved estimate of exposure [27,28,54,55,56]. All four of the naturally occurring aflatoxins have been observed in urine, indicative of exposure. However, our understanding of the toxicokinetics of aflatoxin reveals more quantitative indicators in the form of AFM1 and AF-N7-guanine in urine [57,58,59,60].

A longer-term biomarker of exposure to aflatoxin is found by measuring the concentration of aflatoxin covalently bound to albumin, typically reported as the aflatoxin-lysine equivalent per mg of albumin, also known as the aflatoxin-albumin (AF-alb) adduct [61,62,63,64,65,66]. All three of these biomarkers demonstrate a strong quantitative relationship between intake of aflatoxin and the biological measurement, providing much improved exposure estimates for epidemiological studies (more detail on biomarker development and validation can be found in reviews [26,27,28,54]. As LC-MS/MS technology has advanced [67,68,69,70,71,72], some researchers have discussed reporting aflatoxin-lysine concentrations only [73], and personal communication, avoiding adjustment for albumin by arguing that different assays for albumin provide a slightly different denominator for the AF-alb calculation, and thus, they argue that the use of the albumin adjustment does not refine the exposure estimate. While this observation is valid, it does create difficulty when examining epidemiological studies (and or interventions involving aflatoxin exposure) when trying to compare aflatoxin exposures between studies. A crude estimate can be created with a conversion based on infant age and the serum concentration of albumin. Infants under the age of 1 year have a serum albumin concentration of 0.019–0.048 mg/µL [74]; thus, 1 pg AF-lysine/ul of serum is equivalent to 21–53 pg/mg of AF-alb. After the first year, the normal range of albumin is 0.034–0.042 mg/µL, while after 3 years is 0.035–0.056 mg/µL. This converts a 1 pg/µL measure to a range of 24–29 pg/mg, and 18–29 pg/mg, respectively, with a caveat that this does not include individuals with protein malnutrition, potentially further complicating these estimates.

Where individuals are chronically exposed to aflatoxin, the physiological half-life of the AF-alb adduct can be interpreted such that any given measurement of the adduct provides an integrated estimate of exposure over 2–3 months, smoothing within and across day variations in exposure [41,61,64]. This characteristic means that AF-alb is extremely useful to examine the relationship between exposure to aflatoxin and stunting in regions with a high frequency and variation in aflatoxin contamination of the diet. Typically, more developed world regions, such as Western Europe and North America, will not have detectable AF-alb, while many developing world regions, such as those within sub-Saharan Africa, parts of Central America, and Asia, frequently observe AF-alb in sera [19,24,26,27,65]. In some regions, greater than 90% of individuals, randomly recruited from rural populations in sub-Saharan Africa, have detectable AF-alb, and sometimes across a 3-log range of exposure [75,76,77], while in Canada and the USA, the majority of the samples are below the LOD [27]. Regions such as Brazil and Egypt are thought to be intermediate in exposure [18,19,26,27]. Of note, these historic patterns are likely to shift over time, based on predictive modeling of climate change [78,79,80].

## 5. Associations Between Aflatoxin Exposure and Early Life Stunting

Several animal models have clearly demonstrated the negative effects of aflatoxins on growth, including stunting [81]. Over the last 25 years, several groups have investigated the role of aflatoxin in stunting in human populations. Current reviews tend to report mixed associations, but typically lack critique of the study designs, and a limited understanding of patterns of aflatoxin exposure coupled with a probable variable threshold, discussed later. In this review, Table 1 and Table 2 report several of the published surveys of aflatoxin exposure and stunting, noting, however, that these are representative examples for illustrative purposes and not a systematic review of the evidence. Several studies use stunting as a response outcome with reference to aflatoxin, while others report HAZ (or LAZ) with reference to aflatoxin. Studies are divided into cross-sectional, Table 1 (weaker design), longitudinal, Table 2 (intermediate design), and two interventions, no table (stronger design).

## 6. Cross-Sectional Studies

**Figure 2:**
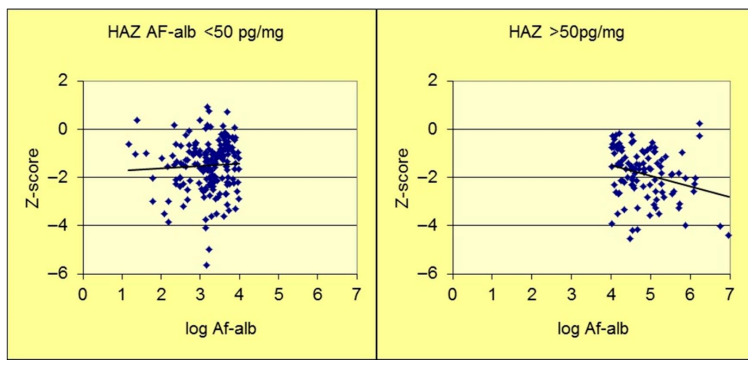
AF-alb versus HAZ scatterplot (adapted from Gong et al., [77]). Scatterplot divided into two sections to highlight a potential point of departure at an AF-alb of about 50 pg/mg. HAZ—Height for Age Z score. AF-alb—aflatoxin-albumin.

The seminal biomarker-driven epidemiological study to assess the relationship between aflatoxin exposure and stunting was a cross-sectional design, conducted in Benin and Togo, that examined anthropometry and serum AF-alb in nearly 500 young children, aged 9–60 months [75,76]. It is important to note that the AF-alb concentration was assessed by ELISA at the University of Leeds (UoL). A strong inverse dose–response relationship was observed between the aflatoxin biomarker and HAZ (*p* < 0.001). Figure 2 identifies a possible threshold below which a log fold variation in AF-alb provides no observable effect on stunting, while a dose–response effect is observed at higher doses. The range of detectable AF-alb in this survey was <3 pg/mg to >1000 pg/mg, and an AF-alb threshold level at perhaps 50 pg/mg. This putative point of deviation has not formerly been determined, and if such a threshold exists, it is likely to vary individually and by study location based on additional gene-environment-nutritional factors for any given population. Linear growth retardation in the early years of life is especially important because the majority of stunted children do not regain a healthy growth trajectory and are likely to suffer irreversible developmental effects. In Benin, the strongest effect of aflatoxin on HAZ was in those three years or less [75,76].

Other cross-sectional studies [50,82,83,84,85] are often reported in the literature reviews, and a modest examination of Table 1 reveals significant design differences. For example, when child growth was examined in The Gambia in relation to aflatoxin exposure [50], the authors reported no statistically significant relationship with stunting. However, the age is significantly greater (6–9 yrs), and notably, the Benin and Togo study [75,76] suggested the effect on growth is more prevalent in those 3 yrs and younger. In the Gambian study, the aflatoxin exposure, again measured as above by ELISA, is high and of concern (some individuals at >100 pg/mg), but does not reach the very high levels in Benin and Togo. To put this into context, no detection of this biomarker was observed in sera from 200 Canadian individuals [18,26], where the limit of detection (LOD) was 3 pg/mg using the same ELISA assay; and less than 1% of individual from a US-NHANES survey of >2000 samples [93] were above the LOD of 0.4 pg/mg using an LC-MS/MS assay. Additionally, the Gambian study involved children who were part of a nutritional supplementation initiative, thus, further complicating any comparisons between aflatoxin and growth. The study in Egypt [82] has been cited in reviews, though it appears to measure AFB1 directly in blood samples. This is not a validated quantitative biomarker of aflatoxin exposure and can only really identify recently exposed versus recently non-exposed individuals; thus, this provides little value in assessing a relationship between typical aflatoxin exposure and stunting.

Aflatoxin exposure measures were added to a pre-existing Kenyan survey involving children and adolescents [83] in an attempt to provide a mechanism for aflatoxin-induced stunting; again, AF-alb concentrations were determined by the previously mentioned ELISA. However, the authors response variable is height rather than HAZ, which has limited value in a cross-sectional study of children over a 12-year age range (6–17 years of age). Younger children consume more per kg bodyweight compared to older adolescents, and thus, an artificial gradient of AF-alb and height across this range may appear statistically significant, but not toxicologically relevant, nor useful. The group also proposed changes in insulin-like growth binding protein-3 (IGFBP-3) as a potential mechanism. IGFBP-3 is a protein that controls the availability of insulin-like growth factor (IGF), and its availability is critical to child growth and development. However, over this same 12-year age range, it would be predicted to change in line with height, so again provides no usable evaluation to suggest a mechanism.

Other cross-sectional studies used an alternate assay for AF-alb; thus, it is important to note that only three labs have conducted cross-comparison studies of their analytical assay for AF-alb. In the first study, sera were analyzed at the UoL by ELISA, the US Center for Disease Control by HPLC-F, and Johns Hopkins University (JHU) by LC-MS/MS; in a second, distinct survey, sera were analyzed by two of the same assays but only involved UoL and JHU. While there was a strong correlation of both data sets, the ELISA, on average, reports levels roughly 5× higher than the LC-MS/MS [67,69]; thus, adjustment for this difference is suggested when comparing studies. A study in Zambia [84] provides a statistically significant relationship between AF-alb biomarker concentration (by LC-MS/MS) and being stunted. Moreover, AF-alb concentrations appear much lower in this survey compared to the Benin and Togo study [75,76]; this apparent discrepancy does not remain after adjustment for differences in analytical approach. In addition, only three of the laboratories have reported any collaborative comparison of standards used. This is particularly critical as the AF-lysine standard used by all laboratories is not commercially available and tends to be synthesized separately in each laboratory. This presents a major hurdle for any review of the evidence of aflatoxin and stunting to overcome and is often ignored in such review papers.

Finally, a cross-sectional study of children aged 1–11 years failed to find an association between AF-alb and stunting [85]. AF-alb was frequent and appeared to be at similar levels to that of the Gambian study [50]. Again, this modest-sized cross-sectional study of children over an extensive 11-year age range is poorly designed to identify causes of stunting, as there are such profound changes over such a disparate physiological range of other growth drivers; and as previously indicated, stunting was most strongly associated with aflatoxin exposure in those three and under [75,76].

In summary, it is difficult to reach any solid conclusions looking at the cross-sectional data presented, not because associations do not agree, but rather, because few studies are even closely related in terms of design, age, and/or use of an exposure tool. Where a strong effect was reported, this was in the 3-year and under category, where a 3-log range of AF-alb occurred.

## 7. Longitudinal Studies

The team at the UoL followed up on their cross-sectional study in Benin and Togo with a novel cohort to conduct a longitudinal survey in 200 Beninese children aged 1–3 years, assessing growth velocity over 8 months [77]. AF-alb was measured in all children on three occasions, start, middle, and end; again, noting each AF-alb measurement is an integrated estimate of exposure over the previous 2–3 months. AF-alb was observed in all children on at least two occasions, and contamination occurred over a 3-log range. Those infants in the highest quartile of AF-alb concentration were observed to be on average 1.7 cm shorter than those in the lowest quartile of AF-alb, after 8 months (*p* < 0.001). This study represents one of the strongest associations between aflatoxin exposure and growth faltering (as stunting) to date.

A longitudinal study followed the growth of Gambian infants from age 6 to 18 months (n = 374), with serum AF-alb measured by ELISA at 6, 12, and 18 months [89]. Anthropometry was obtained at these time points and at 6 months post-blood collection. Inverse relationships were observed between AF-alb and HAZ (*p* = 0.015). AF-alb at 12 months was associated with changes in HAZ between 12 and 18 months of age *p* = 0.003. AF-alb at 6 months was also associated with IGFBP-3 at 12 months (*p* = 0.04), but not for any of the other comparisons they report. A more recent study of Gambian infants identified a relationship between aflatoxin exposure and epigenetic patterns, including changes in methylation of genes involving in part several growth factors [52]. Thus, AF induced modulation of growth by changes in growth factors remains a plausible mechanism of AF induced growth faltering.

A study in Tanzania investigated AF-alb exposure, using ELISA, in relation to growth by following infants for 1 year [87]. Infants (n = 166) were recruited at 6–14 months of age with measures at this baseline and subsequently after 6 and 12 months. Overall AF exposure increased on average as the infants aged in the study; a statistically significant relationship between AF-alb exposure and HAZ was not observed, although a trend was noted. AF-alb detection was frequent but of the order of about a log lower than in the Benin study, perhaps explaining the lack of an observed association.

In a distinct study design, maternal AF-alb was used as a proxy for in utero exposure to aflatoxin [86]. This survey in The Gambia assessed in utero aflatoxin exposure (via two maternal blood draws during pregnancy) in relation to infant growth from birth to age one year, n = 138. Infant anthropometry was recorded from birth and then every 4 weeks up to age 1 year, and these data were compared to maternal average AF-alb measurements. The maternal AF-alb concentration (geometric mean 40 pg/mg, range 5–261 pg/mg) was strongly inversely associated with both weight (*p* = 0.012) and height (*p* = 0.044) gain. The authors predicted that a reduction in maternal AF-alb from 110 pg/mg to 10 pg/mg would lead to a 0.8 kg increase in weight and a 2 cm increase in height by age 1 year. Turner et al. [83] also noted that complementary foods were introduced to 11% of the infants prior to 16 weeks of age, and a 16-week blood draw highlighted that these infants were the only ones to have detectable AF-alb. This additional early life aflatoxin exposure further strongly predicted growth faltering in these infants up to 1 year of age. AF-alb frequency in these Gambian mothers was similar, but adduct concentration was slightly lower than that in Benin infants discussed above. These data suggest that in utero AF may lead to stunting in infancy and that this is further impacted by early introduction of AF contaminated complementary foods; foods that were introduced significantly in advance of WHO recommendations on infant feeding [1]. The relatively small size of this Gambian study, however, limits its generalizability. A separate survey suggested that aflatoxin appears to modulate growth-related gene methylation in infants, where mothers were exposed during pregnancy [53]; thus, supporting early suggestions of a mechanism.

Two studies, one in Bangladesh and one in Ethiopia, found no association between aflatoxin exposure and infant growth, though in both studies the AF-alb adduct was relatively modest [90,91]. The lack of a significant gradient of exposure likely explains the lack of association, as discussed for several cross-sectional analyses. Somewhat surprisingly, a longitudinal survey in Nepal reported statistically significant associations between aflatoxin exposure and infants being stunted, despite very modest aflatoxin exposure in this population [92]. Noting, however, the study is large (n = 1484), providing greater statistical power compared to most, and has multiple exposure measures (four) over the first 18 months post birth. Here, the ranges of exposure are non-detect to only 147 pg/mg, but the geometric means at each collection are all < 2 pg/mg. The AF-lysine here was measured by HPLC with fluorescence (HPLC-F), and no data comparison from that lab in Athens, not CDC, with other methods is reported in the literature. Again, this serves to highlight a major hurdle when comparing studies. Noting where two labs perform their own distinct digestion/extraction and then quantify AF-lys using their own synthesized standards, readers cannot assume the data to be identical just because they both state detection by HPLC-F, which may also have distinct characteristics. However, there is about a 2–3 log variation in biomarker concentration reported in the Nepal study [92]. If HPLC-F and ELISA were providing similar estimates of exposure, then one possible explanation, though speculative, is that Nepalese infants are more sensitive to aflatoxin than sub-Saharan African infants.

The modeling of data in all these longitudinal studies also creates some difficulty. Given the duration and repetition of sampling, it becomes reasonable to think about models that adjust for age and perhaps season. However, age and season are not necessarily independent. Additionally, in some high-risk regions for stunting and aflatoxin exposure, in the first couple of years of life, there are seasonal variations in aflatoxin and food availability. There are also age-dependent changes in intestinal enteropathy and linear growth; thus, in models looking for associations between aflatoxin exposure and stunting, adjustment for age and season may be confounding on both the exposure and the outcome. As these studies evolve to become larger, models that additionally involve subgroup characterization will be valuable. In the Nepalese study, authors also adjusted their statistical model for detect versus non-detect, and the purpose is not clear, given that non-detects were assigned a value.

One study in Malawi involving large numbers of infants followed over a period of 2 years reports a high frequency and range of aflatoxin exposure, and they observe a significant correlation between aflatoxin exposure and stunting. Authors report using pg AF-lysine per ul serum using LC-MS/MS, and crude estimates indicate that 25% of the study population likely exceeds an equivalent of >125 pg/mg (after adjustment) in comparison to the ELISA data [67,69].

A study in Mexico (not included in Table 2) that recruited infants at about 8 months of age and followed for 4 months (including a blood draw for AF-alb) and again at 10 months post-baseline (without a blood draw) [94]. In one model, authors observed a modest improvement in infant growth in relation to aflatoxin at 4 months but not at 10 months, and suggest a hormesis response. Other models in their supplementary section do not see any strong effects. While the detection frequency for AF-alb was high, the concentrations were very modest (0.84 pg/mg, SD 0.7 pg/mg), and the range likely falls within sampling and extraction error when it comes to the ability to accurately estimate exposure using this biomarker. This typical error in exposure estimate is only relevant in studies with very modest AF-alb variation; in general, this remains un-discussed in most aflatoxin papers [18,27]. Here, it creates some uncertainty in interpreting this finding as hormesis. However, aflatoxin-driven hormesis has been suggested in animals [95], and perhaps this modest effect is typically missed in studies with larger aflatoxin distributions. The Mexican study also includes dietary supplementation, and it is unclear if this has influenced the aflatoxin hormesis effect.

Overall, longitudinal studies are somewhat more informative, and studies with larger gradients of aflatoxin exposure are more likely to reveal an effect.

## 8. Intervention Designs

A cluster randomized controlled study in Kenya used 28 intervention and 28 control clusters to examine the effect of aflatoxin intervention on improving infant growth [96]. In the intervention arm, local (predicted contaminated) maize was replaced with aflatoxin-safe maize throughout the study. Women in the fifth to final month of pregnancy were invited to enroll in the study, and infants were assessed at mid-point (age 11 months) and end point (19 months). The main health outcome was HAZ in relation to the study arm at the end point. At the end point, authors claim a significant difference in aflatoxin exposure but not HAZ. Conversion of their Ln transformed AF-alb data reveals geometric means AF-alb of 5.9 pg/mg (95%CI: 5.3, 6.7, n = 436) in the intervention, and 7.5 pg/mg (95%CI: 6.5, 8.5, n = 362) in the control, thus while statistically significant (using *p* < 0.05), toxicologically we would not expect to see differences across this small range. The study seems well designed from an epidemiological standpoint but failed to create a sufficient gradient of exposure across arms. It may also have had a level of aflatoxin exposure that was irrelevant in stunting—see next section. A similar but larger study design in Tanzania aimed to provide aflatoxin-safe flours to 1500 infants from 6 to 18 months and examine the effect of this intervention compared to a control population consuming their normal household flours. The flours consisted of either maize, groundnut, or a mix of maize and groundnut, used in the production of complementary foods in this region [97,98]. Both arms additionally received nutritional and hygiene guidance throughout, with the aim to balance these across arms [99,100]. Several preliminary studies were conducted and revealed (i) a variable and sometimes high level of aflatoxin in typical dietary staples, (ii) a strong acceptance by the infants of the supplied flours, and (iii) a significant reduction in a urinary biomarker (urinary AFM1) after 1 week involving a transition to the aflatoxin-safe flours [101,102]. The study recruited infants into an intervention or control arm (n = 25 hubs for each) based on accessibility to a health center hub. Control and intervention arm hubs were matched for population size and elevation above sea level. The study recruited into both arms monthly over 12 months when infants were <6 months old, and the main study started at 6 months. Mean HAZ was not statistically different between arms at 6 months, and in both arms decreased (HAZ became worse) over the next year, as infants reached 18 months of age. A statistically significant difference in HAZ was not observed at the mid-point, nor at the study end point (though 12-month WAZ and 12- and 18-month MUAC did show statistically significant differences). Food measures for aflatoxin in a subgroup revealed only a modest, albeit statistically significant reduction in maize and groundnut flour contamination [103]. However, in the main study, the use of AF-alb biomarkers (using a novel LC-MS/MS) did not reveal a sufficient gradient in aflatoxin exposure had been achieved between arms [104]. Thus, this second randomized controlled study was ethically designed to target the infant diet and isolate the effect of aflatoxin consumption; however, the natural variation in AF contamination over several years in this region meant the study failed to create an exposure gradient that could test the central hypothesis.

## 9. Complexity of Demonstrating Effective Interventions That Reduce Aflatoxin and Modify Linear Growth

The studies described above that examined the relationship between aflatoxin and either HAZ (or stunting) or attempted to modify the effect on growth via intervention clearly brought up issues on study design and implementation. One highlighted issue is the use of an appropriate biomarker of exposure. In most of the examples discussed, this has been the well-established AF-alb, where in chronically exposed populations a single measurement effectively provides an integrated measure of exposure over several months. At this point, this is the ideal quantitative measure, and while AFM1 in urine could be used, it is more susceptible to shorter-term variation, and urinary AFB1 itself is not useful [18,27,54]. In accepting the AF-alb biomarker, part of the complexity in trying to examine and compare the data within these studies is simply the lack of standardization of two of the analytes typically used, namely a mono-adducted AF-alb as a control material for enzymic digestion, and the AF-lysine residue used for quantitation. Both are typically synthesized in-house, and with a couple of exceptions, no comparative assessments have been made. In one evaluation a specific LC-MS/MS method was compared with an ELISA, and though there was a strong correlation with the two methods, the ELISA typically gave about a 5 x higher adduct measure, possibly as it was influenced by additional AF-residues such as AFG1-lysine and/or incomplete AF-alb digestion that may contain AF-lysine but attached to one or more additional amino acids. Neither residue would be identified by LC-MS/MS due to the specificity of that assay [18,54]. Therefore, where these two assays are used, data adjustment can be attempted. However, there may be alternate assay-specific components that explain this difference, and thus, caution is needed in using this comparison adjustment for other/novel LC-MS/MS approaches. Thus, a concerted effort among multiple players is required to better amalgamate the exposure tools, and this remains a major hurdle. In this respect, the extent of data reporting could be improved. Some studies report GM and 95%CI, others mean and SD, some give ranges, and others, median and IQR. Some of the early studies reported more fully the distribution of data, and this may be valuable as outlined below. Often, we do not know what the distribution of data is, and a simple addition of an appendix showing box and whisker plots or simple distribution bar charts would be useful.

**Figure 3:**
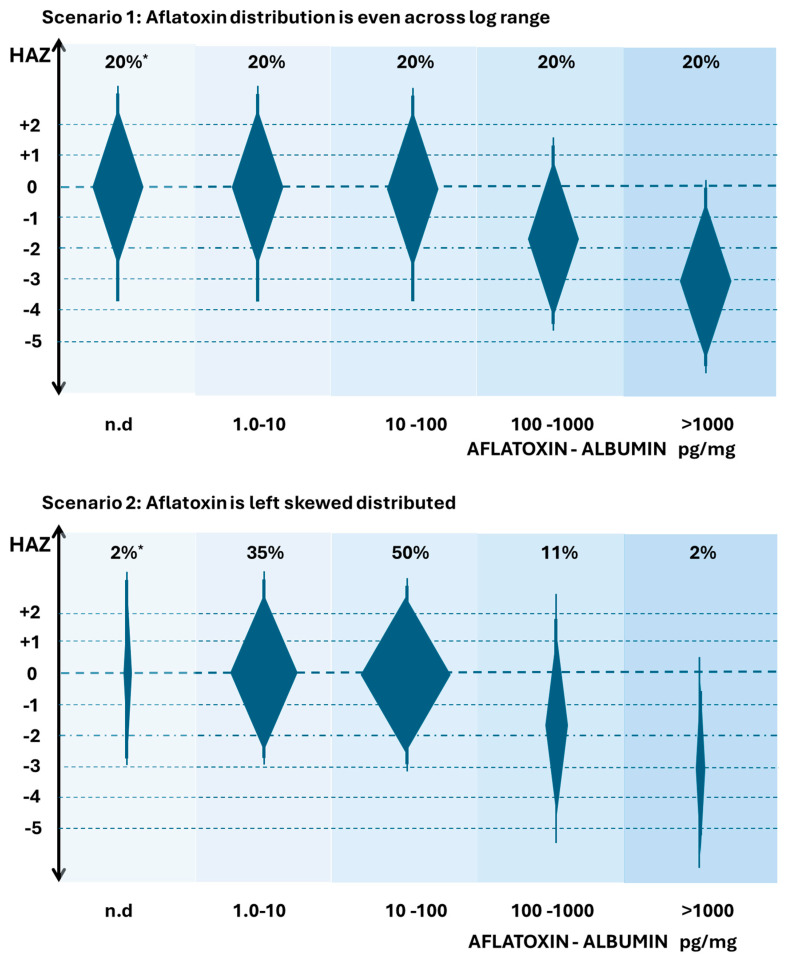
Hypothetical distributions of HAZ in relation to Aflatoxin exposure by group. AF exposure in groups is indicated by aflatoxin-albumin (pg/mg), noting groups log increments. The extended diamond shapes are indicators of HAZ distribution, width is indicative of the suggested distribution of individuals in any group of that aflatoxin exposure, * indicative of the % distribution. All scenarios are based on aflatoxin being a major driver of stunting. Readers are advised not to overinterpret the precision of the data—but rather focus on the changes in distributions. Data are based on the author’s interpretation of studies demonstrating a broad range of aflatoxin exposure in studies on stunting, while the AF-alb values suggested are crudely based on ELISA estimates [75,76,77]. n.d. means non detected.

Figure 3 suggests two possible distributions of aflatoxin exposure (as measured by AF-alb) in relation to an aflatoxin-suggested role in the modification of linear growth. Readers are urged not to overinterpret the minor details of the figures. The hypothetical figures are based on the idea that there is going to be a distribution of HAZ scores in any population, but here, a gradient of AF exposure is highlighted as a major driver of changes in HAZ. The y-axis represents HAZ, and the extended diamond shapes represent possible distributions of HAZ. The x-axis is the AF-alb by group across multiple log increases. The values are based crudely on the ELISA data reported, but readers could envisage adjustment, where available, for other analytical tools as described above. The width of the diamonds is meant to crudely represent the number of individuals in that AF-alb group, which is also indicated by “n” at the top of each group. The widest part of the diamond is at the median HAZ for that group. The background shading of the groups is simply to reinforce the idea of moving to a higher AF-alb group as you go from left to right. Both scenarios suggest there may be a threshold below which aflatoxin has no clear effect. The threshold suggested here is for illustrative purposes, though it is roughly in line with data suggested by surveys in Benin and Togo [75,76,77], where the ELISA was used.

In scenario 1, each exposure group would have roughly equal numbers of infants with the given exposure across a wide range, noting that such a scenario has never been reported in any study to our knowledge. However, if this were the distribution that existed in a high-risk part of the globe, then we could readily observe a large percentage of the population with aflatoxin exposures that have no effect on HAZ, but also a large percentage where aflatoxin had quite a strong effect. In those where aflatoxin had an effect, there is an apparent dose–response component, but also a large amount of variation in HAZ in any given exposure group that is independent and/or additional to the aflatoxin effect. Thus, one could predict that a modest-sized study population may allow a statistically significant association between aflatoxin and HAZ to be observed. It also would indicate that an intervention to reduce AF exposure would be well-positioned to see a reduction in average AF-alb, and if causal, a reduction that then modifies HAZ. This figure also can be used to illustrate that in a region with more modest range of aflatoxin, perhaps only involving exposure in the lower three groups, that a high frequency of exposure could be observed, a gradient of aflatoxin exposure could be observed, and an intervention that successfully reduced exposure could be demonstrated, but crucially, no effect on growth occurs; i.e., a threshold is not reached in the control to create a toxicologically relevant gradient, despite a gradient existing. Many studies report a high frequency of exposure, but perhaps a non-effect of aflatoxin on growth is simply indicative of failing to reach this suggested threshold.

In Scenario 2, a more realistic distribution of AF-alb groups is proposed, a distribution based on a high-risk aflatoxin region, but highly left-skewed. Many studies show this left skew of aflatoxin exposure data, as reviewed [18]. In this scenario, the vast majority of the exposure fails to meet a suggested threshold that would impact HAZ, and the exposure concentrations associated with stunting are restricted to a relatively small percentage of the population. Modestly sized studies would likely not observe a statistically significant effect of aflatoxin on stunting, and the early sections of this review are typically only reporting associations where there was both a large gradient of exposure and a large study size. It could also be expected that intervention studies involving a control arm and an intervention arm would need to be extremely large in order to determine an effect, and that a study with a more modest range of exposure could demonstrate a successful reduction with no impact on HAZ, as described for scenario 1.

**Figure 4:**
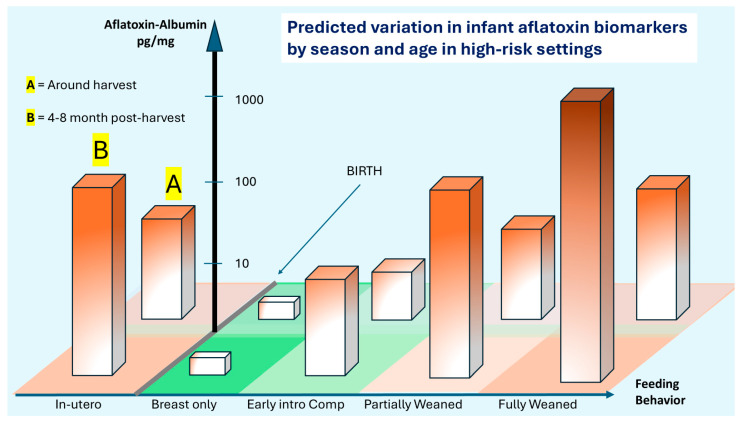
Hypothetical age and seasonal variation in AF-alb in a high-risk setting. Early intro Comp = Early introduction to complementary foods. Proposed scenario of AF exposure estimated by AF-alb measures in early life in a high-risk setting, such as for subsistence or small-hold farmers in sub-Saharan Africa, from in utero through to the infant being fully weaned. The exclusive breastfeeding period is proposed to be highly protective of exposure. The bars are deliberately shaded to indicate that, in any given time frame, there is a range of likely exposures. The probable magnitude of those distributions increases with age, as diets increasingly move away from breast milk and onto high-risk foods, including groundnuts and maize. Seasonal variation is suggested using a crude dichotomy—*back section* (A) harvest is lower risk, and *front section* (B) at 4–8 months post-harvest with a higher risk. Noting that the bars are distributions, some younger infants will have higher levels of AF-alb than older; and some infants in section A will have higher AF-alb than those in section B. A checkered base to the figure is in place simply to assist the reader in identifying groups. Figure is suggested by one interpretation of data from multiple studies, and AF-alb estimates using ELISA [50,75,76,77,86]. Readers are encouraged to focus on the pattern rather than overinterpret the precision of any individual suggested data point in terms of AF-alb values.

The aflatoxin exposure situation is, however, more complex. Figure 4 attempts to additionally portray changes in aflatoxin exposure over time with both ages (food behavior aspect) and seasonality of exposure due to additional accumulation of aflatoxin in food that is in prolonged storage post-harvest [19]. The frequently observed pattern is that aflatoxin accumulates both in the field and in storage, but settings with poor and prolonged storage strongly drive the risk. At birth, infants may be exposed to relatively modest amounts of aflatoxin as the lipophilic toxin can transfer to breast milk if present in the mother’s diet. Typically, as complementary foods are introduced, exposure significantly increases, and during the first two years of life, AF exposure slowly transitions to levels and frequencies seen in older children and adults, and unless the diet and agricultural practices around the diet change, essentially remains similar throughout life, as previously reviewed [18]. Seasonality plays a significant role, typically with the period around harvest having significantly lower (albeit unacceptable levels), and around 4–8 months post-harvest, levels significantly rise. Several studies report on such typical patterns (reviewed by [18]).

Figure 4 serves to visually describe this transition at birth from being relatively aflatoxin-free to being at risk of exposure, but that exposure is dependent upon both diet and the timing of consumption. The units of AF-alb are roughly based on those using ELISA to quantify [50,75,76,77,86]. The figure deliberately uses shading of color to imply a higher exposure at certain stages. The bars should be interpreted as infants having the potential to fall somewhere within the bar, not that all will be at the maximum. Rather, there will be a distribution of data as implied by Figure 3, scenario 2. Thus, in utero exposure can be high, but has quite a potential range, and such high exposures have been associated with infant growth faltering. Then, following birth, there may be quite a significant fluctuation in exposure.

Given the seasonality of exposure, it will be important to understand if there are any critical windows in which exposure to high levels causes greater toxicity. For example, it is plausible to think of two groups of infants that from 6 months to 18 months have the same average level of aflatoxin exposure, but if critical windows exist, is it perhaps more harmful if most of that exposure occurs at 6–10 months of age versus 14–18 months of age; simply based on timing of birth and local harvest timing of the groundnut and maize crops, this remains unexplored. Intervention approaches need to be sufficiently large to account for aflatoxin distribution at any given time, how that varies by season, and how that may be impacted by the timing of the introduction of at-risk foods. The patterns of aflatoxin contamination and, therefore, exposure should not be taken as constant. Annual variation in these patterns can occur and was suggested as a major issue in a recent randomized case–control study [104]. In that pilot study, data indicated significant frequency and levels of exposure, though a suggested change in climate conditions during the 18 months of the main study negatively impacted conditions for aflatoxin production in the control arm; thus, it impacted the ability of the study to show a gradient of exposure between the two arms.

## 10. Recommendations

Aflatoxin has been associated with poor linear growth in several settings and typically occurs where there is a wide gradient of exposure. There are other components that will influence linear growth, including diet and gastrointestinal disease, as well as genetics. When considering diet and hygiene, these may either confound the studies or may be notable components to examine interactions. Such interactions may be important to understand, as modest changes to all three may have a larger-than-expected effect on improving growth. Recommendations from this review include the following:

I. A network is established among aflatoxin researchers to share standards and compare methods, as suggested by Prof David Miller, Dr Mark Sumarah, and other colleagues during one of the Gordon Research Conferences (personal communication)

II. When AF-alb data related (or unrelated) to stunting is generated, authors better describe the distribution of the data. Such data should be shared in a public repository, as it is becoming more of the standard of funders and publication best practice.

III. When designing studies, researchers consider the wide variation in aflatoxin exposures and that aflatoxin biomarker detection is not necessarily toxicologically relevant to stunting as an outcome, unless a suggested threshold is reached. That threshold is not clearly established at this point and may vary by study location and be based on exposure to other drivers of stunting. Establishment of this threshold through statistical studies of previously collected data would be beneficial.

IV. As indicated in Figure 4, researchers consider a larger life course in aflatoxin exposure and its intervention in relation to reducing the burden of stunting. This includes both in utero and over the first few years.

V. Aflatoxin is not the only chemical exposure associated with stunting. Fumonsins are an additional family of mycotoxin that are found in maize in regions that have aflatoxin issues, and may play an additional role, though studies remain limited on co-exposures [87,91].

## 11. Conclusions

Since the seminal studies by Gong and colleagues [75,76,77] that demonstrated strong dose–response relationships between aflatoxin exposure and poor linear growth in infants, many additional studies have been undertaken with mixed results. This review highlights that some at least are due to low exposure, inappropriate biomarkers, and possibly the wrong target population. In studies where an inverse effect between exposure and growth is observed, it could be argued, particularly in the cross-sectional studies, that AF-alb may only be a surrogate measure of malnutrition in an aflatoxin-exposed population; however, the strong evidence in animal studies would counter that argument [81]. Additional cross-sectional or longitudinal studies remain of interest, but causality will only really be established through large-scale intervention studies, where seasonality and other dietary components are well accounted for, as discussed by [104].

The requirement remains “if aflatoxin mitigation efforts in high-risk settings require infant stunting to be demonstrated in order to gain sufficient global traction to be effective in protecting the 100 of millions of people exposed to aflatoxin, larger scale studies covering an extensive in utero through early childhood period are essential”. However, “gold-standard” level of evidence from a randomized trial is often preferred for decision making, we believe that ethical, nonexperimental, interventions are more appropriate for future studies assessing aflatoxin exposures during critical developmental periods. Given the well-documented negative health effects of aflatoxin, mitigating its consumption should be a public health priority.

## Figures and Tables

**Table 1:** Key Cross-sectional studies investigating aflatoxin and either HAZ or being stunted.

Country	Age and n		AF-alb/pg/mg Range and Average		HAZ Stunted *p*-Value		Standard Used ^	Analysis ^^	Ref.
BeninTogo	9–60 mth	479	nd–1000	GM 33	<0.001	n/d	UoL	ELISA	[75,76]
Gambia	6–9 yrs	472	nd–256	GM 22	>0.05	n/d	UoL	ELISA	[50]
Egypt	1–54 mth	46	Serum AFB1	n/r	n/a		Comm *	TLC	[82]
Kenya	7–19 yrs	199	nd–750	GM 111	**		UoL	ELISA	[83]
Zambia	6–24	311	1–315	GM 6	n/d	*p* = 0.02	CDC ***	LC-MS/MS	[84]
Pakistan	1–11 yrs	238	1–56	Med 11	n/d	*p* > 0.05	UoG	HPLC-F	[85]

mth—month, yrs—years, nd—non-detect, n/d—non-determined, GM—Geometric mean. Med—Median, n/r—not relevant ^ Standard used—AF-lysine is not commercially available and was synthesized by individual research groups: UoL—University of Leeds, UK, CDC—Center for Disease Control, USA; UoG—University of Georgia, USA. ^^ ELISA Enzyme-linked Immunosorbent Assay—in-house; TLC—Thin Layer Chromatography; LC-MS/MS—Liquid Chromatography with Mass Spectrometry; HPLC-F—High Performance Liquid Chromatography with Fluorescence, * Comm—commercial purchase—but AFB1 is a measure that exposure has occurred, but cannot be used to quantify exposure. ** height was compared to AF exposure—which is not relevant. *** Uncertain of the source of the standard.

**Table 2:** Longitudinal studies of aflatoxin and stunting 40.4 pg/mg (range 4.8–260.8 pg/mg).

Country	Age and n		AF-alb Range pg/mg and Average	HAZ Stunted *p*-Value		Standard Used ^	Analysis ^^	Ref.
Benin	24–36	200	T1 * T2 nd–1500 5 T3	<0.001	n/d	UoL	ELISA	[77]
Gambia	<12	138	Preg 5–261 40 16 Wk 5–30 9	<0.05	n/d	UoL	ELISA	[86]
Tanzania	6–14	166	T1 n/d 5 T2 n/d 13 T3 n/d 24	>0.05	n/d	UoL	ELISA	[87]
Nepal	12–36	85	T1 n/d 4 T2 n/d 3 T3 n/d 4	>0.05	n/d	JHU	LC-MS/MS	[88]
Gambia	6–18	374	T1 n/d 3 T2 n/d 25 T3 n/d 52	<0.05	n/d	UoL	ELISA	[89]
Bangladesh	7–36	228	T1 <0.1–6 Mn 1 T2 <0.1–6 2 T3 <0.1–66 3 T4 <0.1–127 4	Overall *p* > 0.05		JHU	LC-MS/MS	[90]
Ethiopia	6–35	102	75th percentile < LOD	>0.05	n/d	UoC	LC-MS/MM	[91]
Nepal	3–22	1484	Preg <0.4–147 2 T1 <0.4–25 1 T2 <0.4–54 1 T3 <0.4–85 1 T4 <0.4–128 1	*p* = 0.003	*p* = 0.005	UoG	HPLC-F	[92]
MALAWI	<30	241	AF-lysine pg/uL	<0.05	n/d	JHU	LC-MS/MS	[73]

T = time point for multiple collections, Preg—during pregnancy, * Average of three > 98% of Ts had detectable AF-alb. nd—non-detect, n/d—no data. Average is the Geometric mean unless stated. Mn—arithmetic mean. ^ Standard used—AF-lysine is not commercially available and was synthesized by individual research groups: UoL—University of Leeds, UK; UoG—University of Georgia, USA; JHU—Johns Hopkins University, USA. ^^ ELISA Enzyme-linked Immunosorbent Assay—in-house; LC-MS/MS—Liquid Chromatography with Mass Spectrometry; HPLC-F—High Performance Liquid Chromatography with Fluorescence.

## Data Availability

No new data were created or analyzed in this study.
